# Discrimination of p53 immunohistochemistry-positive tumors by its staining pattern in gastric cancer

**DOI:** 10.1002/cam4.346

**Published:** 2014-10-30

**Authors:** Koji Ando, Eiji Oki, Hiroshi Saeki, Zhao Yan, Yasuo Tsuda, Gen Hidaka, Yuta Kasagi, Hajime Otsu, Hiroyuki Kawano, Hiroyuki Kitao, Masaru Morita, Yoshihiko Maehara

**Affiliations:** 1 Department of Surgery and Science, Graduate School of Medical Sciences, Kyushu University3-1-1 Maidashi, Higashi-ku, Fukuoka City, Fukuoka, 812-8582, Japan; 2 Department of Molecular Oncology, Graduate School of Medical Sciences, Kyushu University3-1-1 Maidashi, Higashi-ku, Fukuoka City, Fukuoka, 812-8582, Japan

**Keywords:** Gastric cancer, immunohistochemistry, mutation analysis, p53, staining pattern

## Abstract

Immunohistochemistry staining of p53 is a cheap and simple method to detect aberrant function of p53. However, there are some discrepancies between the result of immunohistochemistry staining and mutation analysis. This study attempted to find a new definition of p53 staining by its staining pattern. Immunohistochemistry staining of p53 and *TP53* gene mutation analysis were performed in 148 gastric cancer patients. Also SNP-CGH array analysis was conducted to four cases. Positive staining of p53 was observed in 88 (59.5%) tumors. Tumors with positive p53 staining showed malignant features compared to negative tumors. Mutation of *TP53* gene was observed in 29 (19.6%) tumors with higher age and differentiated type. In positive p53 tumors, two types could be distinguished; aberrant type and scattered type. With comparison to *TP53* gene mutation analysis, all the scattered type had wild-type *TP53* gene (*P* = 0.0003). SNP-CGH array showed that scattered-type tumors had no change in the structure of chromosome 17. P53-scattered-type staining tumors may reflect a functionally active nonmutated *TP53* gene. In interpretation of p53 immunohistochemistry staining, distinguishing p53-positive tumors by their staining pattern may be important in gastric cancer.

## Introduction

Gastric cancer still has the highest morbidity rate and the second highest mortality rate in Asian countries. Though many new remedy for gastric cancer have discovered, advanced gastric cancers are still difficult to treat. To conquer gastric cancer, understanding the gastric carcinogenesis may be important.

One of the causes for gastric cancer is *Helicobacter pylori* infection. It is reported that *H. pylori* counteracts the function of tumor suppressor p53 1,2. Also, the infection causes *TP53* gene mutation 3. p53, the guardian of genome, is involved in many cellular function; cell cycle, apoptosis, restoration of DNA, aging, and angiogenesis 4,5. And breakdown of tumor suppressor p53 pathway results in the development of malignancies. Studying p53 is still important in gastric cancer.

To detect the aberrant p53, sequencing of the mutant *TP53* gene is a general method. After the discovery of the first mutation in *TP53* gene 6, the study on *TP53* gene was done vastly. The results from these studies revealed that ∼75% of the mutant *TP53* gene had missense mutation 7. This finding was well-characterized for *TP53* gene mutation as other tumor suppressor genes such as *APC* or *BRCA1/2* frequently have nonsense mutation or frameshift mutation. By investigating the impact of each mutation on p53 function, we may understand the role of *TP53* gene mutation in carcinogenesis.

To find the aberration of *TP53* gene in everyday clinic, a simple and cheap method is used. That is immunohistochemistry staining of p53. In normal condition, p53 is unstable because its half-life is no longer than 20 min owing to cleavage through Mdm2 8, which cannot be detected by staining. When p53 is aberrant, the nucleus of the cell is stained and aberrant p53 can be detected.

Many studies are done on immunohistochemistry staining of p53. In lung cancer, meta-analysis study revealed that positive immunohistochemistry staining of p53 is an independent prognostic factor 9,10. Immunohistochemistry staining of p53 is a useful tool to understand the biology of cancer.

However, the result of immunohistochemistry staining of p53 reflects the aberrant function of p53 or not is controversial. Some reports showed discrepancies between the results of immunohistochemistry and those of mutation analysis 11–13.

Here, we report a new discrimination on immunohistochemistry staining of p53 by its staining pattern. This discrimination may reflect the result of mutation analysis. Also, we introduced SNP-CGH array method to demonstrate the accuracy of this discrimination.

## Material and Methods

### Tissue samples

This study included 148 unselected Japanese patients with primary gastric cancer. All of the patients underwent gastrectomy between 1994 and 2006 at the Department of Surgery and Science, Graduate School of Medical Sciences, Kyushu University Hospital, Fukuoka, Japan. Informed consent was obtained from all patients, and those who did not agree to the study were excluded. A thorough histological examination was carried out with hematoxylin- and eosin-stained tissue preparations, and classification was made according to the general rules established by the Japanese Gastric Cancer Association 14. No patients who were treated preoperatively with cytotoxic drugs were included in this study.

### Immunohistochemical staining of p53

Formalin-fixed, paraffin-embedded tissue specimens were used for immunohistochemical staining. A paraffin block contained both cancerous and adjacent noncancerous tissue, and cancerous tissue that invaded the deepest area of the stomach wall was selected in all cases. Sections 5 *μ*m thick from paraffin-embedded blocks were deparaffinized in xylene and rehydrated in a graded series of ethanol. Procedures for immunohistochemical staining have been described previously 15. The sections were pretreated with autoclaving at 121°C for 15 min in 0.01 mol/L citrate-buffered saline (pH 6.0) for antigen retrieval. Endogenous peroxidase activity was blocked by incubation with 3% H_2_O_2_ for 30 min at room temperature. The sections were incubated with 10% normal goat serum for 1 h to block nonspecific binding of the immunological reagents. After incubation with mouse monoclonal antibodies against p53 (Clone DO-7; Dako Cytomation, Glostrup, Denmark) at 4°C overnight, streptavidin–biotin complex and horseradish peroxidase were applied, and reaction products were visualized using the Histofine SAB-PO (M) immunohistochemical staining kit (Nichirei, Tokyo, Japan), according to the manufacturer's instructions. The peroxidase labeling was developed by incubation of the sections in diaminobenzidine tetrahydrochloride for 1 min. Finally, nuclear counterstaining was done using Mayer's hematoxylin solution. Two blinded observers (K. A. and Y. Z.) independently examined the immunostained sections. When >10% of nuclear-stained cancer cells were included in the section, the tumor was considered to be p53 positive 16,17.

### DNA preparation

DNA was extracted as described previously 18,19. Briefly, the frozen samples were incubated in a lysis buffer (0.01 mol/L Tris-HCl, pH 8.0; 0.1 mol/L ethylenediaminetetraacetic acid (EDTA), pH 8.0; 0.5% sodium dodecyl sulfate) containing proteinase K (100 mg/mL) at 37°C for 2 h. The samples were extracted twice in phenol, then once in phenol/chloroform and once in chloroform. Following ethanol precipitation, the samples were diluted in TE (0.01 mol/L Tris-HCl, pH 8.0; 0.01 mol/L EDTA, pH 8.0) buffer.

### The *TP53* gene mutation analysis

The *TP53* gene, exon 5 to exon 9 including exon–intron junctions, were amplified by polymerase chain reaction (PCR) using “p53 primers” (Nippon Gene) and Ex Taq DNA polymerase with 3′ exonuclease activity (TaKaRa Bio Inc., Tokyo, Japan). The PCR products were purified and used as templates for cycle sequencing reactions with Big Dye Terminator Cycle Sequencing Kit Ver.1.0 (Applied Biosystems, Foster City, CA). Mutations found in a PCR product were verified by reverse sequencing and reconfirmed in two independently amplified PCR products.

### SNP-CGH analysis

Four surgically resected gastric cancer specimens and their corresponding noncancerous tissues were genotyped by using 1,140,419 autosomal SNPs (HumanOmni1-Quad BeadChip; Illumina Inc., San Diego, CA). Copy number variation was analyzed with GenomeStudio V2009.1 (Illumina Inc.) as described previously 20. Two transformed parameters, the log-normalized intensity ratio (log *R* ratio) and B allele frequency, were plotted along the entire genome for all SNPs on the array in the single sample analysis mode.

### Statistical analysis

Statistical analysis was performed by using JMP 9.0 software (SAS institute, Cary, NC). The *χ*^2^ test, Fisher's exact test, and one-way ANOVA were used as appropriate. A *P* < 0.05 was considered significant.

## Results

### Patients and tumors

Of the 148 patients, 65.5% (*n* = 97) were male and 34.5% (*n* = 51) were female. The mean age was 63.4 ± 11.6, ranging from 29 to 86.

### p53 staining in gastric cancer

**Figure 1:**
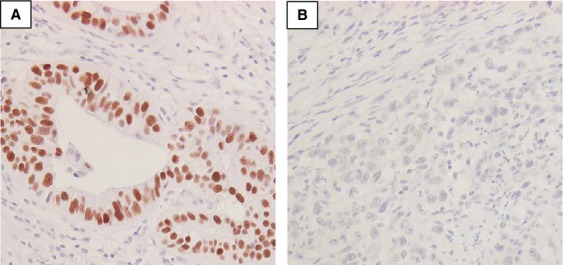
Staining pattern of p53 in gastric cancer. (A) Positive case. Staining of p53 is seen in the nuclei (magnification, 400×). (B) Negative case. No staining of p53 is seen in the nuclei (magnification, 400×).

It is widely recognized that, tumors with aberrant function of p53, p53 can be stained immunohistochemically 21. Consistent with previous studies, the staining of positive p53 was seen in the nuclei of tumor cells (Fig.1A) 16,22. On the other hand, negative p53 staining tumors showed no signal of p53 (Fig.1B). Of 148 tumors, 88 (59.5%) had positive staining of p53. With clinicopathological analysis (Table1), p53-positive tumors had more lymph node and liver metastases compared with tumors with negative p53 staining (*P *=* *0.02, *P *=* *0.02, respectively). Also, more vascular involvement was seen in aberrant p53-positive tumors (*P *=* *0.03). p53-positive tumors tended to have deeper invasion (*P *=* *0.06). Tumors with positive staining of p53 had malignant features.

**Table 1:** P53 expression and clinicopathological factors in gastric cancer patients.

	p53 expression (%)		
Factors	Negative (n = 60)	Positive (n = 88)	*P*-value
Age (mean ± SD)	63.5 ± 11.2	63.4 ± 12.1	0.95
Gender			
Male	38 (63.3)	59 (67.1)	0.64
Female	22 (36.7)	29 (32.9)	
Differentiation			
Differentiated	25 (41.7)	35 (39.8)	0.81
Undifferentiated	35 (58.3)	53 (60.2)	
Vascular involvement			
V0	36 (60.0)	44 (50.0)	0.03*
V1	16 (26.7)	24 (27.3)	
V2	8 (13.3)	12 (13.6)	
V3	0 (0.0)	9 (9.1)	
Lymphatic involvement			
Ly0	19 (31.7)	26 (29.6)	0.21
Ly1	17 (28.3)	19 (21.6)	
Ly2	18 (30.0)	23 (26.1)	
Ly3	6 (10.0)	20 (22.7)	
Depth of invasion			
M, SM	10 (16.7)	6 (6.8)	0.06
MP, SS, SE, SI	50 (83.3)	82 (93.2)	
Lymph node metastasis			
Negative	26 (43.3)	23 (26.1)	0.02*
Positive	34 (56.7)	65 (73.9)	
Liver metastasis			
Negative	60 (100)	83 (94.3)	0.02*
Positive	0 (0)	5 (5.7)	
Stage			
I + II	32 (53.3)	37 (42.1)	0.17
III + IV	28 (46.7)	51 (57.9)	

M, mucosa; SM, submucosa; MP, muscularis propria; SS, subserosa; SE, penetration of serosa; SI, invasion of adjacent structures.

* *P* < 0.05.

### TP53 gene mutation analysis in gastric cancer

Of 11 exons that exist in *TP53* gene, exon5 to exon8 is a major spots for mutation; R175H, G245S, R248Q, R248W, R249S, R273C, R273H, and R282H 23. In this study we investigated the mutations of *TP53* gene in exon5 to exon9. As shown in Table2, of 148 tumors, 29 (19.6%) had mutant *TP53* gene. Of those, six tumors (20.7%) showed nonsense mutation and other 23 tumors (79.3%) showed missense mutation. *TP53* gene mutations were found more frequently in differentiated-type tumors than in undifferentiated type (*P *=* *0.009). This result was consistent to previous report 24. Relation with other clinicopathological factors showed that, *TP53* gene mutations were found in higher age and positive vascular involvement tumors (*P *=* *0.02, *P *=* *0.01, respectively).

**Table 2:** *TP53* gene status and clinicopathological factors in gastric cancer patients.

	*TP53* gene status (%)		
Factors	Wild (n = 119)	Mutant (n = 29)	*P*-value
Age (mean ± SD)	62.5 ± 12.1	67.2 ± 9.3	0.02*
Gender			
Male	75 (63.0)	22 (75.9)	0.18
Female	44 (37.0)	7 (24.1)	
Differentiation			
Differentiated	42 (35.3)	18 (62.1)	0.009*
Undifferentiated	77 (64.7)	11 (37.9)	
Vascular involvement			
V0	70 (58.8)	10 (34.5)	0.01*
V1	32 (26.9)	8 (27.6)	
V2	14 (11.8)	6 (20.7)	
V3	3 (2.5)	5 (17.2)	
Lymphatic involvement			
Ly0	34 (28.6)	11 (37.9)	0.06
Ly1	25 (21.0)	11 (37.9)	
Ly2	36 (30.2)	5 (17.2)	
Ly3	24 (20.2)	2 (6.9)	
Depth of invasion			
M, SM	11 (9.2)	5 (17.2)	0.23
MP, SS, SE, SI	108 (90.8)	24 (82.8)	
Lymph node metastasis			
Negative	41 (34.4)	8 (27.6)	0.47
Positive	78 (65.6)	21 (72.4)	
Liver metastasis			
Negative	116 (97.5)	27 (93.1)	0.28
Positive	3 (2.5)	2 (6.9)	
Stage			
I + II	53 (44.5)	16 (55.2)	0.3
III + IV	66 (55.5)	13 (44.8)	

M, mucosa; SM, submucosa; MP, muscularis propria; SS, subserosa; SE, penetration of serosa; SI, invasion of adjacent structures.

* *P* < 0.05.

### Relation between p53 immunohistochemistry and TP53 gene mutation

Then relation between p53 immunohistochemistry staining and *TP53* gene mutation analysis was investigated. Twenty percent of p53-negative tumors had mutant *TP53* gene (12/60 tumors) and 19% of p53-positive tumors had mutant *TP53* gene (17/88 tumors) (*P* = 0.91). This result showed that there is no significant relation between p53 immunohistochemistry staining and *TP53* gene mutation.

We then sought for a new definition for p53 immunohistochemistry staining pattern.

### TP53 gene mutation in p53-negative tumors reflect both wild-type and nonsense mutation

First we examined the *TP53 gene* mutation status in 60 tumors with p53-negative staining. Of 60 tumors, 48 (80%) tumors had wild-type *TP53 gene*. And as shown above, 6 (10%) tumors had nonsense mutation. The rest six tumors had missense mutation. Negative staining of p53 may reflect wild-type *TP53* gene or nonsense mutation which needs sequence analysis.

### Two staining pattern of p53-positive tumors; scattered type and aberrant type

**Figure 2:**
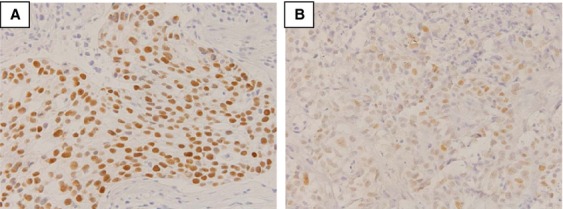
Staining pattern of “aberrant type” and “scattered type” in positive staining group. (A) Aberrant type. Widespread staining of all nuclei of the tumor sample is seen (magnification, 400×). (B) Scattered type. Staining of single cells scattered throughout the tumor sample (magnification, 400×).

In this study, 88 tumors had positive staining of p53. In these tumors, 10% or more nuclei were stained with p53. According to previous report 25, we also found that in these positive tumors, two subtypes could be distinguished by its staining pattern and percentage of stained nuclei; “aberrant type” and “scattered type.” Aberrant type (Fig.2A) had widespread staining of 70% or more nuclei in the sample. Of 88 tumors, this type was seen in 63 (71.6%) tumors. On the other hand, “scattered type” (Fig.2B) had staining of single cells throughout the tumor sample. In these tumors 20–50% of nuclei were stained. Of 88 tumors, 25 (28.4%) tumors were this type. Compared to p53-negative tumors, “scattered type” tumors had deeper invasion and tend to have more lymph node metastases (Table3). This result showed that p53-negative tumors and “scattered type” tumors have different features. There was no relation with clinicopathological factors between scattered type and aberrant type (data not shown). However, a significant relation was observed with *TP53* gene mutations (Table4). Interestingly, all scattered-type tumors were wild-type *TP53* gene. And all mutant *TP53* gene tumors, which were missense, had aberrant-type staining of p53 (*P* = 0.0003). It seemed that “scattered type” reflects wild-type p53.

**Table 3:** Clinicopathological factors in gastric cancer patients with negative p53 and scattered type.

	p53 expression (%)		
Factors	Negative (*n* = 60)	Scattered (*n* = 25)	*P*-value
Age (mean ± SD)	63.5 ± 11.2	64.9 ± 12.6	0.62
Gender			
Male	38 (63.3)	15 (60.0)	0.77
Female	22 (36.7)	10 (40.0)	
Differentiation			
Differentiated	25 (41.7)	7 (28.0)	0.22
Undifferentiated	35 (58.3)	18 (72.0)	
Vascular involvement			
V0	36 (60.0)	14 (56.0)	0.19
V1	16 (26.7)	9 (36.0)	
V2	8 (13.3)	1 (4.0)	
V3	0 (0.0)	1 (4.0)	
Lymphatic involvement			
Ly0	19 (31.7)	9 (36.0)	0.11
Ly1	17 (28.3)	3 (12.0)	
Ly2	18 (30.0)	6 (24.0)	
Ly3	6 (10.0)	7 (28.0)	
Depth of invasion			
M, SM	10 (16.7)	0 (0.0)	0.006*
MP, SS, SE, SI	50 (83.3)	25 (100)	
Lymph node metastasis			
Negative	26 (43.3)	6 (24.0)	0.08
Positive	34 (56.7)	19 (76.0)	
Stage			
I + II	32 (53.3)	9 (36.0)	0.14
III + IV	28 (46.7)	16 (64.0)	

M, mucosa; SM, submucosa; MP, muscularis propria; SS, subserosa; SE, invasion of adjacent structures.

* *P* < 0.05.

**Table 4:** Scattered staining reflects *TP53* gene wild type in p53 positive gastric cancer.

	p53 expression (%)		
	Scattered (*n* = 25)	Aberrant (*n* = 63)	*P*-value
*TP53* gene status			
Wild (*n* = 71)	25 (100)	46 (73.0)	0.0003
Mutation (*n* = 17)	0 (0)	17 (27.0)	

### SNP-CGH array analysis revealed a different pattern of chromosome 17 between “aberrant” and “scattered” type

**Figure 3:**
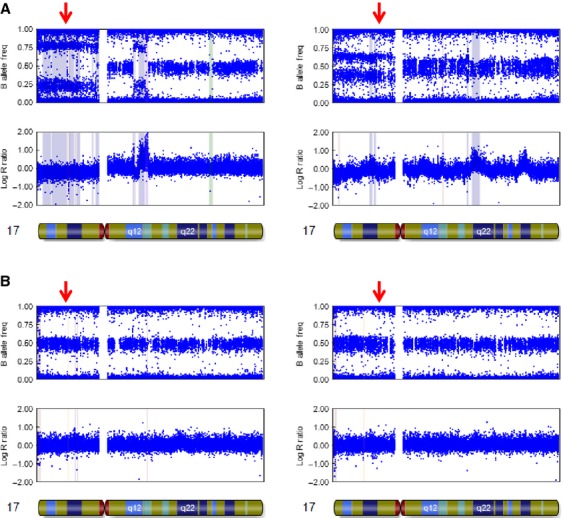
SNP-CGH array analysis of chrmosome17 in aberrant type and scattered type. (A) Analysis in aberrant type. Abnormal chromosome is seen. Especially, aberrant allele is observed in short arm. (B) Analysis in scattered type. The chromosome 17 is normal. The red arrow shows the locus of p53.

For further analysis on p53, we performed SNP-CGH array analysis in four gastric cancer samples. Two “aberrant type” and two “scattered type” were analyzed. For aberrant type, one *TP53* gene wild-type tumor (Fig.3A left) and one mutant-type tumor (Fig.3A right) were analyzed. With regard to chromosome 17, data from two “aberrant type” specimen showed that there was a deflection in the log R ratio (Fig.3A). In these two specimens, including the *TP53* gene wild-type tumor (Fig.3A left), the heterozygous state split into two clusters in the B allele frequency was also observed, especially in the short arm containing the p53 locus. These results showed p53 was aberrant from the chromosomal state in “aberrant type.”

On the contrary, in “scattered type” no deflection in the log *R* ratio was observed. And, the heterozygotes were clustered around *t*_0.5_ in the B allele frequency (Fig.3B), which was also observed in the noncancerous tissue (data not shown). As previously shown, *TP53* gene is wild type in these two tumors.

These results suggested that “scattered type” staining had normal structure of p53 from the chromosomal state.

## Discussion

To date, p53-positive tumors have been considered to have aberrant p53 function. However, there was some discrepancy between the result from immunohistochemistry and sequence analysis. In this study, we reevaluated the p53-positive tumors to two groups by its staining patterns. This evaluation showed us that even in p53-positive tumors, there were tumors with normal p53 function. Staining pattern may be important in interpreting the result of p53 immunohistochemistry.

Immunohistochemistry of p53 is said to be a cheap and rapid method to detect aberrant p53 function. In general, tumors with more than 10% positively stained cancer cells nuclei were defined as positive 16,17,26. In this study, 59.5% tumors had positive staining of p53. With clinicopathological analysis, p53-positive tumors had malignant features compared to negative tumors; more lymph node metastases and more liver metastases, which were consistent with previous reports 15–17,27. Immunohistochemistry of p53 might interpret the biology of the tumor.

On the other hand, about 20% of the tumor had mutant *TP53* gene in this study. This result was consistent to previous reports 19,24. With clinicopathological analysis, tumors with mutant *TP53* gene were frequently observed in higher age and differentiated cases. Differentiated gastric cancers are more frequently observed in older patients and follows multifocal atrophic gastritis, which is accompanied by intestinal metaplasia or dysplasia. In fact, multistep gastric carcinogenesis including a sequence of events that begins with *H. pylori*-induced superficial gastritis, progressing toward chronic atrophic gastritis, intestinal metaplasia, dysplasia, and finally cancer 28. It is reported that *H. pylori* infection induces point mutations of *TP53* in 52% of gastritis 29. Also, Wei et al. reported that reported that *H. pylori* accelerates ubiquitination and proteasomal degradation of p53 in gastric epithelial cells 30. *H. pylori* infection and p53 may act in a synergistic fashion in gastric carcinogenesis 31. All these reports suggest that *TP53* gene mutation has an important role in gastric carcinogenesis.

Immunohistochemistry staining of p53 has been proposed as an alternative to gene analytical method. However, in our study, only distinguishing positive or negative staining of p53 had no effect in detecting the mutation status of *TP53* gene, similar to previous reports 11,13.

Kaserer et al. reported a study on staining patterns of p53 in colorectal cancer 25. They found that in positive p53 tumors, there were two types in staining pattern of p53; wide spread staining of all nuclei of the tumor cells and staining of single cells scattered throughout the tumor sample. In this study, we also classified p53-positive tumors in two groups according to their report; aberrant type and scattered type. “Scattered type” had staining of single cells throughout the tumor sample. In these tumors 20–50% of nuclei were stained. These tumors had deeper invasion and more lymph node metastases compared to p53-negative tumors which showed that “scattered type” tumors are different to p53-negative tumors. Also, we found that the entire “scattered type” staining group had wild-type TP53 gene. Even in p53-positive tumors, there were tumors with p53 normal function. Yemelyanova et al. reported an immunohistochemical and nucleotide sequence analysis of p53 in ovarian carcinomas 32. They found that combining two immunohistochemical labeling patterns associated with *TP53* mutations (0% and 60–100% positive cells), correctly identified a mutation in 94% of cases (*P* < 0.001). They concluded that immunohistochemical analysis can be used as a robust method for inferring the presence of a *TP53* mutation in ovarian carcinomas. In our study, we focused on “scattered type” and wild-type *TP53*. In Yemelyanova's report there were two cases with 20–50% positive cells, which is same to our “scattered type,” and these cases had wild-type *TP53* gene. This result may reflect our results; “scattered type” tumor has wild-type *TP53* gene.

From SNP-CGH array analysis, this finding was much clearer. The development of high-density SNP genotyping technology for genomic profiling represents a further advance, because simultaneous measurement of both signal intensity variations and changes in allelic composition makes it possible to detect chromosomal events 33. Data from two “scattered type” p53 staining specimens (Fig.3B) and all normal samples (data not shown) indicated no chromosomal alterations for the entire chromosome 17, especially on the p53 locus. This result also demonstrated that “scattered type” had normal p53 function.

Then why normal p53 can be stained; normal p53 should have short half-life that cannot be stained. Hall and Lane reported that the reason for normal p53 expression might be that wild-type p53 protein accumulates in response to spontaneous genetic errors occurring at a higher frequency in the tumor than in normal surrounding tissue 12. This “scattered type” staining of p53 may rather reflect an accumulation of wild-type p53 protein as a result of either a response to DNA damage, alterations in the normal degradation process, or the stabilization of the gene product by an interaction with viral or cellular proteins 34,35.

However, a question remains; why are there still wild-type *TP53* gene tumors in “aberrant type?” In this study we found 46 tumors with wild-type *TP53* gene in 63 “aberrant type” tumors, which is a quite ratio. The staining pattern does not match with “scattered type.” We think that “aberrant type” represents a clonal expansion of cells accumulating p53. Clonal expansion of p53 accumulating cells in the presence of a wild-type *TP53* gene has been observed previously 36. This was shown to represent stabilization of the protein by mechanisms other than inactivating mutations 36,37. The wild-type tumors in “aberrant type” may reflect this mechanism. From SNP-CGH array analysis in *TP53* gene wild type of “aberrant type” tumor (Fig.3A left) may support this discussion. Even though this tumor had wild-type *TP53* gene, its chromosome 17 had aberrant structure. In “scattered type,” chromosome 17 did not have aberrant structure. A further study is required to clarify the “aberrant type.”

Our study showed that positive staining of p53 has two types by its staining patterns in gastric cancer. “Scattered type” may reflect normal p53. In interpretation of p53 immunohistochemistry staining, discriminating p53-positive tumors by their staining pattern is important in gastric cancer.
